# Buruli Ulcer: a Review of the Current Knowledge

**DOI:** 10.1007/s40475-018-0166-2

**Published:** 2018-09-28

**Authors:** Rie R. Yotsu, Koichi Suzuki, Rachel E. Simmonds, Roger Bedimo, Anthony Ablordey, Dorothy Yeboah-Manu, Richard Phillips, Kingsley Asiedu

**Affiliations:** 1 School of Tropical Medicine and Global Health, Nagasaki University, Nagasaki, Japan; 2 Department of Dermatology, National Center for Global Health and Medicine, Tokyo, Japan; 3 Department of Dermatology, National Suruga Sanatorium, Shizuoka, Japan; 4 Department of Clinical Laboratory Science, Faculty of Medical Technology, Teikyo University, Tokyo, Japan; 5 Department of Microbial Sciences, School of Bioscience and Medicine, University of Surrey, Surrey, UK; 6 Department of Medicine, VA North Texas Healthcare System, Dallas, TX USA; 7 Division of Infectious Diseases, University of Texas Dallas Southwestern, Dallas, TX USA; 8 Department of Bacteriology, Noguchi Memorial Institute for Medical Research, University of Ghana, Legon, Ghana; 9 Kumansi Centre for Collaborative Research in Tropical Medicine, Kwame Nkrumah University of Science and Technology, Kumasi, Ghana; 10 Department of Control of Neglected Tropical Diseases, World Health Organization, Geneva, Switzerland

**Keywords:** Buruli ulcer, *Mycobacterium ulcerans*, Mycolactone, Non-tuberculous mycobacterial disease, Skin neglected tropical diseases, Skin NTDs

## Abstract

**Purpose of the Review:**

Buruli ulcer (BU) is a necrotizing and disabling cutaneous disease caused by *Mycobacterium ulcerans*, one of the skin-related neglected tropical diseases (skin NTDs). This article aims to review the current knowledge of this disease and challenges ahead.

**Recent Findings:**

Around 60,000 cases of BU have been reported from over 33 countries between 2002 and 2017. Encouraging findings for development of point-of-care tests for BU are being made, and its treatment is currently in the transition period from rifampicin plus streptomycin (injection) to all-oral regimen. A major recent advance in our understanding of its pathogenesis has been agreement on the mechanism of action of the major virulence toxin mycolactone in host cells, targeting the Sec61 translocon during a major step in protein biogenesis.

**Summary:**

BU is distributed mainly in West Africa, but cases are also found in other parts of the world. We may be underestimating its true disease burden, due to the limited awareness of this disease. More awareness and more understanding of BU will surely contribute in enhancing our fight against this skin NTD.

## Introduction

**Fig. 1:**
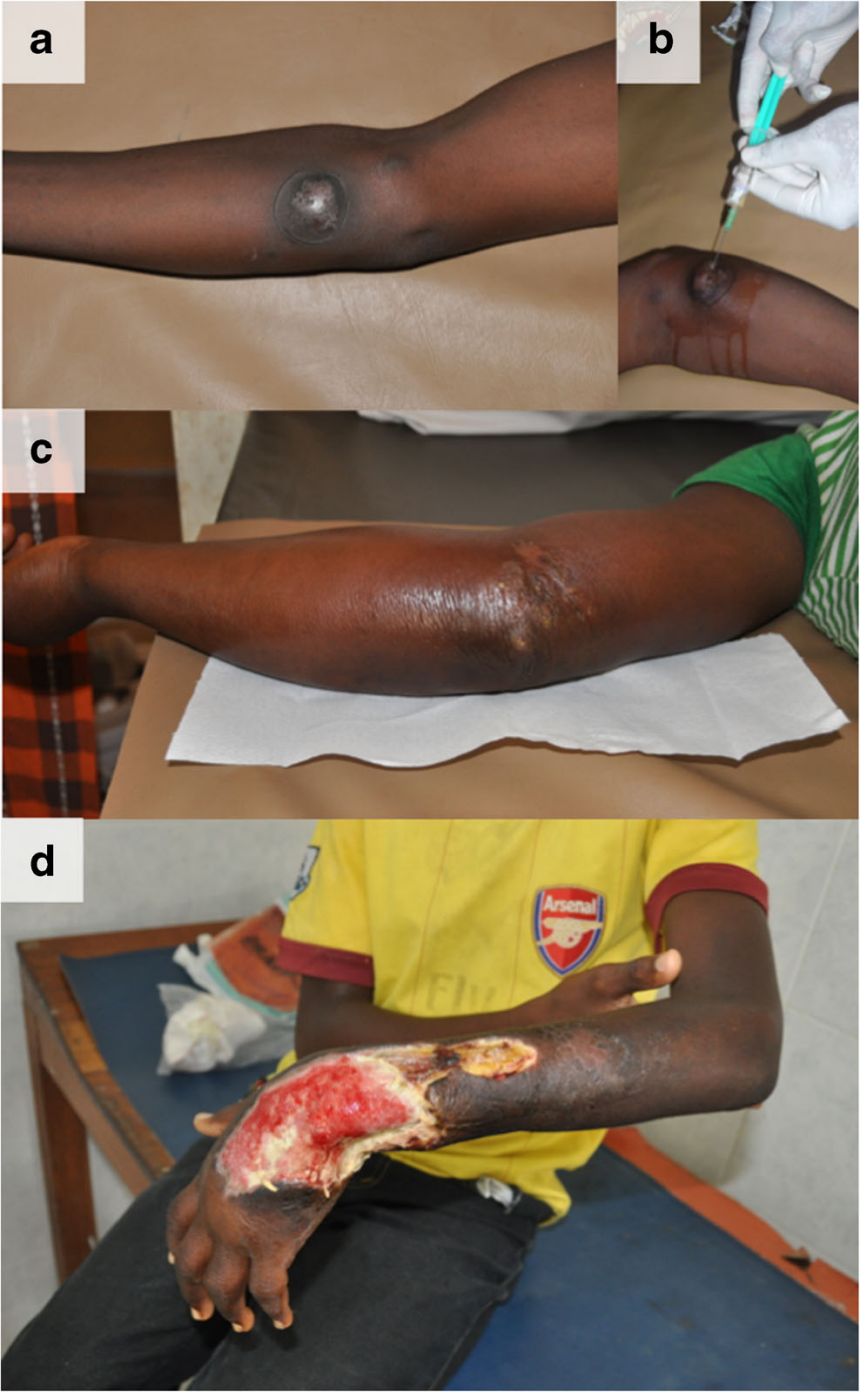
Clinical presentation of Buruli ulcer, **a** nodule stage. **b** Pale yellowish pus was aspirated by the fine needle aspiration and sent for PCR confirmation. **c** Edematous stage. This stage is usually associated with redness, swelling, as well as considerable pain. **d** Typical ulcer observed in Buruli ulcer. Deep, undermining of the wound edges, with thick necrotic tissue affecting the limb and the joint of a child

Buruli ulcer (BU) is a necrotizing cutaneous disease caused by the bacterium, *Mycobacterium* (*M*.) *ulcerans*, which is classified by the World Health Organization (WHO) as one of the skin-related neglected tropical diseases (skin NTDs) [1•, 2]. Clinically, BU starts with a papule, nodule, plaque, or edematous lesion that eventually progress to extensive skin ulceration (Fig. 1). Remarkably given the extent of tissue loss, the lesion is usually painless or only with limited pain. Unlike other mycobacterial diseases, a unique aspect of BU is that the pathology of the disease can be ascribed to its lipid-like and diffusible exotoxin, mycolactone, and not the organism itself. The majority of cases are seen in West Africa and in other tropical countries; however, the disease has also been reported in countries with subtropical and temperate climates. Imported cases have been reported from non-endemic countries, and this calls for more awareness among healthcare practitioners globally. While antibiotic therapy is available and usually effective, patients with severe forms or delayed therapy could be left with life-long disabilities and deformities. Early detection and treatment is currently the only measure to prevent deleterious consequences, especially in a disease that often affects children. Yet, several unanswered questions remain which would be key to controlling this disease, including identification of the route(s) of transmission and some aspects of its pathogenesis. In this article, we review updated knowledge on epidemiology, clinical presentation, and management of this disease and future challenges.

## Update on Epidemiology

BU was first described in Australia by MacCallum in 1948 [3, 4]. Currently, over 33 countries worldwide—including West Africa, Central and South America, and the Western Pacific—report cases of BU. The West African countries of Côte d’Ivoire, Ghana, and Benin are the top three endemic countries accounting for approximately 73% of the total BU cases reported globally [5] (Table 1). The highest numbers of cases reported from these three countries were 2679 cases in 2009, 1157 cases in 2004, and 1203 cases in 2007, respectively. A decrease in reported cases from these countries is however observed in recent years, for unclear reasons, including possible under-diagnosis or under-reporting of cases. Nigeria, on the other hand, started to report cases of BU since 2006 with gradual increase in their reported cases [5], which may be an indication of wider distribution of the disease in countries in this region. In Africa, about 48% of cases occur in children aged under 15 years [6], the most vulnerable population, which increases the public health importance in tackling this disease.

**Table 1:** Number of cases of Buruli ulcer by country, 2002 to 2017 (WHO data: http://apps.who.int/gho/data/node.main.A1631)

	2002	2003	2004	2005	2006	2007	2008	2009	2010	2011	2012	2013	2014	2015	2016	2017	Total
Australia	32	14	34	47	72	61	40	35	42	143	105	74	89	111	186	283	1368
Benin	565	722	925	1045	1195	1203	897	674	572	492	365	378	330	311	312	267	10,253
Cameroon	132	223	914	265	271	230	312	323	287	256	160	133	126	133	85	No data	3850
Central African Republic	No data	No data	No data	No data	No data	No data	3	No data	No data	No data	No data	No data	No data	No data	No data	No data	3
Congo	102	180	235	53	370	99	126	147	107	56	38	6	No data	No data	No data	No data	1519
Côte d’Ivoire	750	768	1153	1564	1872	2191	2242	2679	2533	1659	1386	1039	827	549	376	344	21,932
Democratic Republic of the Congo	17	119	487	51	74	340	260	172	136	209	284	214	192	234	175	91	3055
Equatorial Guinea	No data	No data	No data	3	No data	No data	No data	No data	No data	0	No data	No data	No data	No data	No data	No data	3
Gabon	No data	No data	43	91	54	32	53	41	65	59	45	59	47	43	39	45	716
Ghana	853	737	1157	1005	1096	668	986	853	1048	971	632	550	443	275	371	538	12,183
Guinea	No data	157	146	208	279	No data	80	61	24	59	82	96	46	72	72	98	1480
Japan	No data	No data	1	1	1	3	2	5	9	10	4	10	7	4	2	6	65
Liberia	No data	No data	No data	No data	No data	No data	No data	No data	No data	No data	21	8	No data	105	No data	219	353
Nigeria	No data	No data	No data	No data	9	No data	No data	24	7	4	40	23	65	114	235	259	780
Papua New Guinea	13	18	31	No data	No data	26	24	8	5	8	No data	No data	3	11	16	5	168
Sierra Leone	No data	No data	No data	No data	No data	No data	1	No data	No data	28	No data	No data	No data	No data	No data	No data	29
South Sudan	568	360	4	24	38	8	3	5	4	No data	No data	No data	No data	No data	No data	No data	1014
Togo	96	38	800	317	40	141	95	52	67	52	51	37	67	81	83	62	2079
Uganda	117	10	7	72	5	31	24	3	No data	No data	No data	No data	No data	No data	No data	No data	269
Total	3245	3346	5937	4746	5376	5033	5148	5082	4906	4006	3213	2627	2242	2043	1952	2217	61,119

Interestingly, unlike many other tropical diseases, BU is also seen in countries outside the tropical climate zone, including Australia and Japan. In Australia, cases are especially concentrated in the bay area of the Victoria region, in the south-eastern part of the country [7]. The situation in Australia seems to be evolving, with sharp increase in number of cases from a previous average of 100 to 186 cases in 2016 and 283 cases in 2017 [5]. In Japan, a total of 66 cases since 1981 have been reported to date, with no specific geographical distribution [8]. Several cases are reported by the same medical institutions, suggesting that the awareness of healthcare practitioners is the key for the diagnosis.

There have been sporadic case reports of imported cases from endemic regions, for example, from Australia to the USA and from Angola to Germany [9, 10]. A case diagnosed in the Netherlands, with polymerase chain reaction (PCR) investigation, revealed the causative bacteria to be the same strain as the cases in Japan—*M. ulcerans* subsp. *shinshuense* [11]. The patient had a travel history to the Shan Dong Province of China where the latitude is above 30° N, and where there have been no previously described BU cases. BU occurrence at such high latitude is rare and had only been reported in Japan, suggesting that *M. ulcerans* subsp. *shinshuense* is more adapted to environments at higher latitudes than strains of the classical *M. ulcerans* lineage in tropical and subtropical climate zones [11]. With accelerating globalization and with existence of case reports from new sites, awareness of this disease among healthcare practitioners is needed more widely to enable diagnosis and appropriate treatment, and also to understand its true distribution.

## Uncovering the Mode of Transmission

The mode of transmission of BU remains poorly understood. As cases are concentrated in areas with proximity to slow moving or stagnant bodies of water (ponds, swamps, marshes, backwaters, dams, artificial lakes), the current hypothesis is that the disease is transmitted from these environments to humans [12]. This is further supported by the clinical presentation of the disease: the lesions are often distributed on the exposed areas of the body including the limbs and the face. There is no evidence to support the possibility of human-to-human transmission of BU [13].

Investigation on potential reservoirs of *M. ulcerans* is ongoing. The slow growth rate of *M. ulcerans* (doubling time > 48 h) coupled with its low density poses special difficulties in the recovery of the bacteria in culture from environmental samples. Ideal decontamination protocols capable of inhibiting all contaminating fast-growing bacteria and fungi existing with *M. ulcerans* is required [12, 14]. However, an improved culture technique has recently been proposed that may overcome this [15]. Currently, real-time PCR targeting *M. ulcerans*-specific DNA sequences (the insertion sequences IS*2404* and IS*2406*, and genes present on the mycolactone-encoding plasmid such as KR-B and ER) is the technique used most frequently in surveys of environmental specimens [12, 16]. Although the presence of DNA does not provide definite proof of the presence of living bacteria, identification of *M. ulcerans* DNA has been successful in environmental samples ranging from water filtrates, soil, biofilms, fish, frogs, snails, crayfish, insects, to other invertebrates [14, 17–31]. Reports of successful culture of live bacteria from environmental samples are much rarer but have been reported from samples of an aquatic insect (Hemiptera or water strider) as well as from moss and aulacode (greater cane rat) feces [14, 15, 32].

Remarkably, evidence from West African countries and Australia suggest that the mode of transmission may be different in tropical and temperate climates. For instance, while there is some evidence for mosquitoes as a potential passive vector for *M. ulcerans* in Australia [18, 33–35], there is less consistent support from studies in Benin, which did not detect *M. ulcerans* DNA in mosquito species [36] whereas a study in Cameroon did [37]. A few experimental laboratory studies also failed to confirm the implication of mosquitoes as biological agents for the transmission of *M. ulcerans* [38, 39•]. In a study by Djouaka et al., ingestion of *M. ulcerans* was observed during the larvae stage of the mosquitoes but not during both pupae and adult stages, revealing the low ability of infected or colonized mosquitoes to vertically transmit *M. ulcerans* to their offspring [40]. Interestingly, a recent study by Wallace et al. demonstrated and proposed that a micro-puncture in the skin from any cause, whether from insect bites, injuries, or other natural means, has the potential to inject *M. ulcerans* from the environment into the skin and generate ulcers [39•]. Such a mechanism would also be sufficient to explain the potential role of biting water insects such as Naucoridae and Belostomatidae [12], which have received a lot of attention as potential vectors in West African countries.

There may be several routes of transmission in BU as the disease occurs in a range of different epidemiological settings and geographic regions, as well as some role for various living organisms acting as reservoirs and as vectors.

## Risk Factors for Buruli Ulcer

In addition to living in proximity of potentially contaminated water sources, other postulated risk factors for acquiring BU are related to aquatic environment; for instance, receiving insect bites near a river, swimming in or wading through a river, bathing with water from open borehole, and farming [33, 41–44]. Younger age (< 15 years), poor pre-existing wound care, and failure to wear protective clothing are other presumed risk factors, as well as lack of use of mosquito nets [43–45]. A study in Australia demonstrated the use of insect repellent was associated with reduced risk while reporting of mosquito bites was associated with increased risk [33]. Seasonality is also suspected, and some studies report the risk of acquiring the disease increases during the wet season [16]. However, not all infected persons manifest the disease, and spontaneous healing has been observed to occur [46–49].

Besides these socio-demographic, environmental, or behavioral factors, there may also be some genetic host susceptibility factors to BU. Some genetic host susceptibility factors are observed in other mycobacterial diseases including natural resistance-associated macrophage protein-1 (*NRAMP-1*), HLA-DR, vitamin D_3_ receptor, mannose-binding protein, interferon-gamma (IFN-*Γ*) receptor, tumor necrosis factor alpha (TNF-ɑ), interleukin (IL)-1ɑ and IL-1β and their receptor antagonists, and IL-12 [50]. A study in Benin revealed history of BU in a family member was associated with increased risk (OR, 5.5 (95% CI, 3.0–10.0), *p* < 0.001) [51]. A similar observation has been made in other studies. However, data on possible genetic host factors is still limited. One study from Ghana has shown a genetic polymorphism in the *SLC11A1* gene to play a role in susceptibility to develop BU, with an estimated 13% population attributable risk [52]. Currently, preventative measures for BU are not exactly known and a better understanding of these genetic risk factors may enable the development of effective and efficient measures in the future.

## Pathogenesis—More Discoveries in Mycolactone

Pathogenesis of BU relies on mycolactone, a polyketide-derived macrolide that is synthesized by the *M. ulcerans* bacteria [53]. It is synthesized by giant polyketide synthetases coded in a 174-kb plasmid pMUM001, with possible involvement of other genes on the bacterial chromosome [53, 54]. Three major biological functions of mycolactone have been identified including cytotoxicity, immunosuppression, and analgesic effects, which correspond well to the characteristic features of the disease, i.e., extensive deep ulceration with thick yellowish necrotic tissue and undermining, paucity of local inflammatory response, and no or limited pain.

It is now widely accepted that the major cellular target of mycolactone is the Sec61 translocon [55]. This molecular machine sits at the interface between the cytosol and the endoplasmic reticulum (ER) and is essential for the translocation of around 30–50% of proteins that must cross this membrane. Such proteins are involved in vital processes that drive cell-cell communication such as the immune response. Hence, Sec61-dependent loss of cytokines [56, 57], cellular receptors [57], and antigen presentation molecules [58] has now been shown to underpin the immunosuppression seen in the disease.

Inhibition of Sec61 also explains mycolactone’s cellular toxicity. Single amino acid substitutions in the *Sec61A1* gene (encoding the α subunit of the translocon) have been shown to confer resistance to the cytotoxic effects of mycolactone [57]. Furthermore, an unbiased screen for resistance mutations in the human genome identified Sec61A1 substitutions alone. Recently, the molecular mechanism linking this to cell death was shown to involve the mislocalisation of Sec61 substrates into the cytosol [56], leading to uncontrolled cellular stress and Bim/Bcl2-mediated apopotosis [59].

Mycolactone has also been shown to inhibit mTOR activity [60], and causes hyper-activation of WASP, members of a family of scaffold proteins transducing variety of signals into dynamic remodeling of the cell’s actin cytoskeleton [61]. Furthermore, it activates type 2 angiotensin II receptors, resulting in hypoesthesia through potassium-dependent hyperpolarization of neurons. This may explain the painlessness or minimal pain associated with BU lesions [62], yet other studies suggest that other functions, including potential cytotoxity effect against the Schwann cells, account for this phenomenon [63]. It is not surprising that several pathways are present, making mycolactone a multifunctional cytotoxin given the pathological features of BU. Mycolactone is indeed an interesting molecule, and further understanding of its function may extend beyond understanding of BU pathogenesis.

It is important to note that mycolactone production is not limited to *M. ulcerans*. Indeed, there is a whole family of mycolactone-producing mycobacteria (MPM) including certain strains of *M. marinum*, *M. pseudoshottsii*, *M. liflandii*, and *M. xenopi* [64, 65]. Slight differences in the modular arrangement of genes on pMUM001 mean that they each produce different congeners of mycolactone [55].

## Diagnosis—New Diagnostic Tools?

Currently, BU diagnostic confirmation is done through detection of *M. ulcerans* DNA using PCR of IS*2404*, IS*2606*, and ER. Other methods for confirmation of BU include microscopic detection of acid-fast bacilli in lesions, cultures, and histopathology. WHO target is for over 70% of reported cases to be PCR confirmed [6]. However, many of the endemic areas do not have an easy access to facilities to conduct these tests and thus case confirmation continues to be a challenge.

New diagnostic tools are currently under development not only for early detection of cases, especially ones that could be used at field level, but also for case management. These include the loop-mediated isothermal amplification (LAMP) test [66, 67], thin-layer chromatography for the detection of mycolactone [68], and antigen detection assays [69]. Field applicable formats of these tests are under development to provide rapid and sensitive diagnostic tests close to BU patients.

In the absence of a point-of-care diagnostic test, clinical diagnosis can be made in endemic areas with some degree of reliability given the distinctive clinical features of this disease. A recent study by Eddyani et al. reported that attending clinicians’ diagnosis of BU had a high sensitivity of 92% (95% CI, 85–96%) compared against the reference diagnosis made by the expert panel in Benin [70]. Nonetheless, there is a wide range of differential diagnoses of skin ulcers, and more understanding of the disease distribution encountered in BU hospitals/clinics of endemic regions is necessary [70–72]. The accelerating development of technology and communication networks, the use of teledermatology and artificial intelligence, in the future, may enhance diagnosis of BU and other skin ulcers in the field [1, 2, 73].

## Treatment with No Injection

In 2004, based on in vitro findings and pilot-clinical studies, the WHO recommended a combination of rifampicin (10 mg/kg orally once daily) and streptomycin (15 mg/kg intramuscularly once daily) for 8 weeks as the first-line therapy for BU [74]. Before this time, surgery was the core treatment, believed to be the most effective, but it was associated with prolonged hospitalization, high cost, and high recurrence rate (6–21.5%) [75–78]. Introduction of this antibiotic treatment has substantially changed the management and led to better outcomes. Cure was achieved at a much higher rate, and fewer patients needed amputation [79].

However, not only is streptomycin injection associated with significant nephrotoxicity and ototoxicity, it required daily visits to healthcare centers or hospitalization, depriving patients time and opportunities to conduct their daily activities. The impact could be more pronounced in children—the most affected population—affecting their education. This also affected early case detection as this created fear among patients and families and prevented them from seeking care at healthcare facilities [80].

Currently, all oral antibiotic therapy for BU is being investigated [81]. Data from a randomized control trial and several systemic case series studies [82–84], suggest that an all-oral antibiotic combination of rifampicin and clarithromycin (7.5 mg/kg twice daily) would be effective [6]. Other rifampicin-based combinations are also being studied, for example, rifampicin plus ciprofloxacin, clarithroymycin, moxifoxacin, ethambutol, amikacin, or azithromycin in Australia [85, 86] and rifampicin, clarithromycin, plus levofloxacin in Japan [87]. Besides antibiotic types, we may also need to revisit the dosage and treatment duration, which have largely been decided on the basis of experience and therefore lack in evidence [81].

Paradoxical reaction is the worsening of symptoms during effective antibiotic treatment, which is another characteristic feature of BU. This is due to a recovered host inflammatory response to *M. ulcerans* and to the dying bacteria with fall in the mycolactone levels [88]. This phenomenon is reported to occur at the rate of between 1.9 and 26% [83, 85, 86, 89–91] and has previously been mistaken for recurrence or relapse in some cases. Paradoxical reactions might also have led to unnecessary surgical interventions due to mistakenly perceived treatment failure. Recently, a randomized controlled trial in Benin investigated the possible effects of delaying the decision to operate at 14 weeks rather than at standard 8 weeks would have any effect [92•]. Their results showed that delaying the decision to operate led to decreased rate of surgery and reduced median time of hospitalization and wound care, without any effect on occurrence of functional impairment [92•]. This provides a new insight into the treatment of BU, that we may have been misinterpreting the need of surgical intervention at an inappropriate time-point when paradoxical reaction is likely to happen. On the other hand, we also need to appreciate the benefits of surgery that it may have on severe cases. More assessment and development of guidelines, algorithms, or training materials are needed to facilitate the surgical interventions in BU for better outcomes.

## Importance of Wound Management in Buruli Ulcer

Wound management is an important pillar in the treatment of BU but often goes neglected. Ulcers can take months and years to heal, even after successful treatment with antibiotics under inadequate care. Large scars and contractures leading to functional impairment may further happen due to prolonged ulceration [93, 94]. Nonetheless, there are a number of challenges in settings where the disease is endemic to achieve a high standard of wound management from hygiene to availability of materials.

Good wound management is required not only for faster and sequelae-free epithelialization of ulcers but also for increased quality of life of patients. BU lesions were previously noticed to be associated with no or limited pain, but recent studies have revealed that many patients actually experience pain after the start of treatment [72, 95, 96]. This pain is most felt during wound dressing change when in many settings, gauze is the only dressing type being used [97]. Gauze is inexpensive and readily available, but the disadvantages are that it adheres to the wound bed not only causing pain during dressing change, but also may impair the dermal regeneration.

There has been an extensive development in wound management techniques and materials in recent years as the market is growing with the increase in number of patients with skin ulcers from non-infectious causes, e.g., diabetes and peripheral arterial diseases, in developed countries. Although cost may be an issue in using them, they may result in shortening the wound healing time, ultimately contributing to cost reduction. Some innovative approaches have been piloted for management of BU wounds, including use of absorbent form dressing HydroTac^®^ (Harmann, Heidenheim, Germany) and negative-pressure wound therapy V.A.C. Therapy System^®^ (Kinetics Concepts Inc. (KCI), San Antonio, USA) with successful outcomes [98, 99]. More of these experiences are needed to be accumulated, together with cost analysis, to address the long-term benefit of such treatments. Development of similar, more field-friendly wound management techniques and materials with reduced cost are anticipated. In addition, as standards of wound management are heterogeneous between healthcare providers and between institutions [97], development of training materials and courses are also another needed intervention.

## Prevention

Some prevention against BU has been observed with *Mycobacterium bovis* bacillus Calmette-Guérin (BCG) vaccination, but the results are controversial [100]. Vaccines specific to *M. ulcerans*, targeting a mycolyl transferase (antigen 85A) of the bacteria, are also being tested [101], but there is as yet no established definite measure for preventing the disease. At present, the only preventative measures that can be taken are to avoid risky behaviors (swimming, fishing, agricultural works, etc.) and environmental contacts in endemic areas.

## Conclusions

Introduction of antibiotic treatment in 2004, with rifampicin and streptomycin, has made a big change in management of BU and ultimately changed the lives of those affected. Since then, fewer patients need surgical interventions and amputations are no longer a common treatment option. The search for better and easier treatment options is in progress. Additionally, a better understanding of the mode of transmission of the disease and development of field-friendly rapid diagnostic tools are essential in order to enhance our fight against BU. Mycolactone is a unique toxin secreted by *M. ulcerans* which could also be a target substance in control of this disease. In recent years, the disease group of skin NTDs has been gaining more attention due to its potential for effective integration of their control. This current trend could bring about another big change in the control of this disabling disease.
